# Nonsurgical spinal decompression system traction combined with electroacupuncture in the treatment of multi-segmental cervical disc herniation: A case report

**DOI:** 10.1097/MD.0000000000028540

**Published:** 2022-01-21

**Authors:** Qing Xu, Xuewen Tian, Xintong Bao, Dongren Liu, Fanshuo Zeng, Qiangsan Sun

**Affiliations:** a Department of Rehabilitation Medicine, The Second Hospital, Cheeloo College of Medicine, Shandong University, Jinan, Shandong, China; b Department of Rehabilitation Medicine, Shandong Sports Rehabilitation Research Center, Jinan, Shandong, China; c Shandong Sport University, Jinan, Shandong Province, China; d Department of Sports Medicine, Shandong Sports Rehabilitation Research Center, Jinan, Shandong, China; e Medical Imaging Department, Shandong Sports Rehabilitation Research Center, Jinan, Shandong, China.

**Keywords:** articular, biomechanical phenomena, cervical disc herniation, electroacupuncture, intervertebral disc displacement, physical therapy modalities, range of motion, traction

## Abstract

**Rationale:**

With the spread of computers and mobile phones, cervical spondylosis has become a common occupational disease in clinics, which seriously affects the quality of life of patients. We used a nonsurgical spinal decompression system (SDS) combined with physical therapy electroacupuncture (EA) to treat a case of mixed cervical spondylosis caused by multi-level cervical disc herniation, and we achieved satisfactory results.

**Patient concerns:**

A 44-year-old Caucasian Asian woman presented with neck pain and numbness on the left side of the limb. MRI showed the patient's C3–C7 segment cervical disc herniation, and the flexion arch of the cervical spine was reversed.

**Diagnosis:**

The patient was diagnosed with a mixed cervical spondylosis.

**Interventions:**

The patient received a month of physical therapy (SDS traction combined with EA).

**Outcomes:**

Before and after treatment: VAS score of neck pain decreased from 8 to 0; Cervical spine mobility returned to normal; The grip strength of left hand increased from 7.5 kg to 19.2 kg; Cervical curvature index changed from −16.04% to −3.50%; the physiological curvature of the cervical spine was significantly restored. There was no dizziness or neck discomfort at 6 month and 1 year follow-up.

**Lessons subsetions:**

SDS traction combined with EA is effective for the treatment of cervical disc herniation and can help restore and rebuild the biomechanical balance of the cervical spine.

## 1 Introduction

Cervical disc herniation is a common spinal disease in the clinic, which seriously affects the quality of life of patients and poses a heavy economic burden to individuals and society.^[1]^ In recent years, with the widespread use of mobile phones and computers and the increased living pressure of contemporary people, the incidence of cervical disc herniation has shown a younger trend. Understanding the pathogenesis, effective prevention, and treatment of cervical disc herniation are particularly important. This paper reports a patient with multisegmental cervical disc herniation who was effectively treated with a non-surgical spinal decompression system (SDS) and electroacupuncture (EA).

## 2 Case presentation

**Figure 1:**
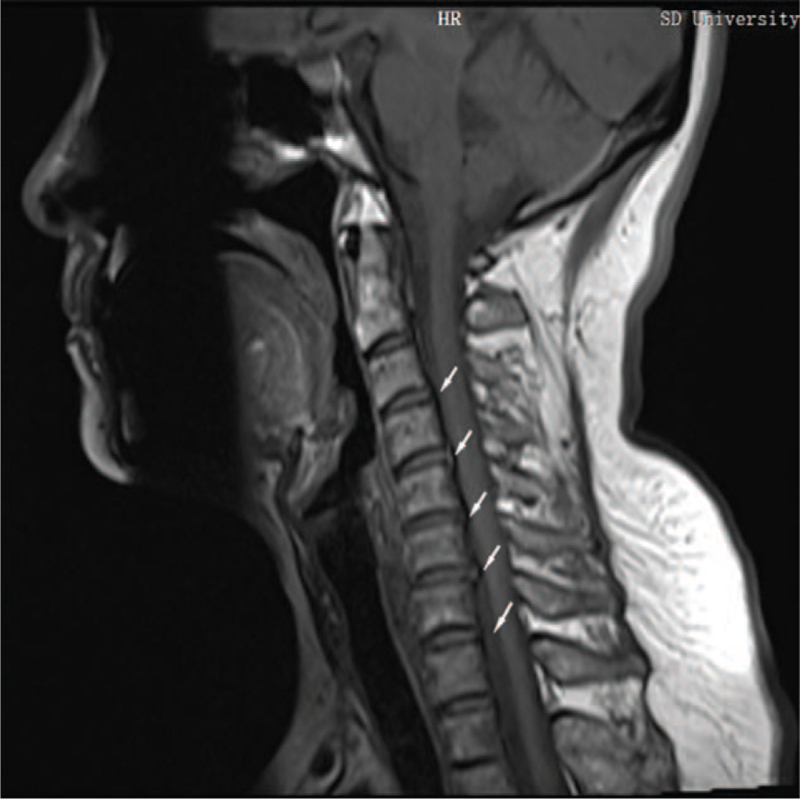
T1 weighted sagittal image of cervical spine before treatment. Note: The white arrows indicate the location of the cervical disc herniation and the location of the dural and spinal cord compression. Fig. 1-4. Sagittal image of cervical spine before treatment and after treatment.

**Figure 2:**
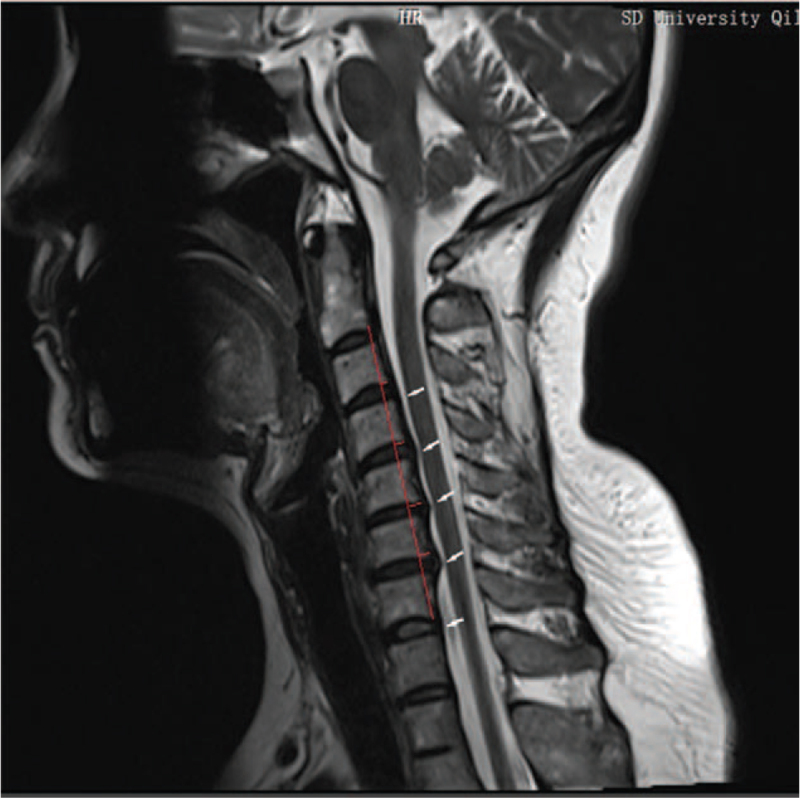
T2 weighted sagittal image of cervical spine before treatment. Note: The white arrows indicate the location of the cervical disc herniation and the location of the dural and spinal cord compression, and the red line measures the flexion angle of the cervical spine.

A 44-year-old Caucasian Asian woman was diagnosed with a mixed cervical spondylosis. Her main complaints were dizziness, stiff neck and shoulders, and numbness in the left limb. MRI showed a C3-C7 segment cervical disc herniation (Figs. 1 and 2). No abnormalities were found on brain MRI; therefore, cerebrovascular diseases were excluded. The patient received conservative treatment (massage, short-wave therapy, intravenous drugs, etc.) in the hospital for 2 weeks, but no obvious effect was observed. She rejected the suggestion of surgical treatment and came to our center for assistance. The patient had no history of family genetic diseases, infectious diseases, hypertension, or cardiovascular diseases.

**Figure 3:**
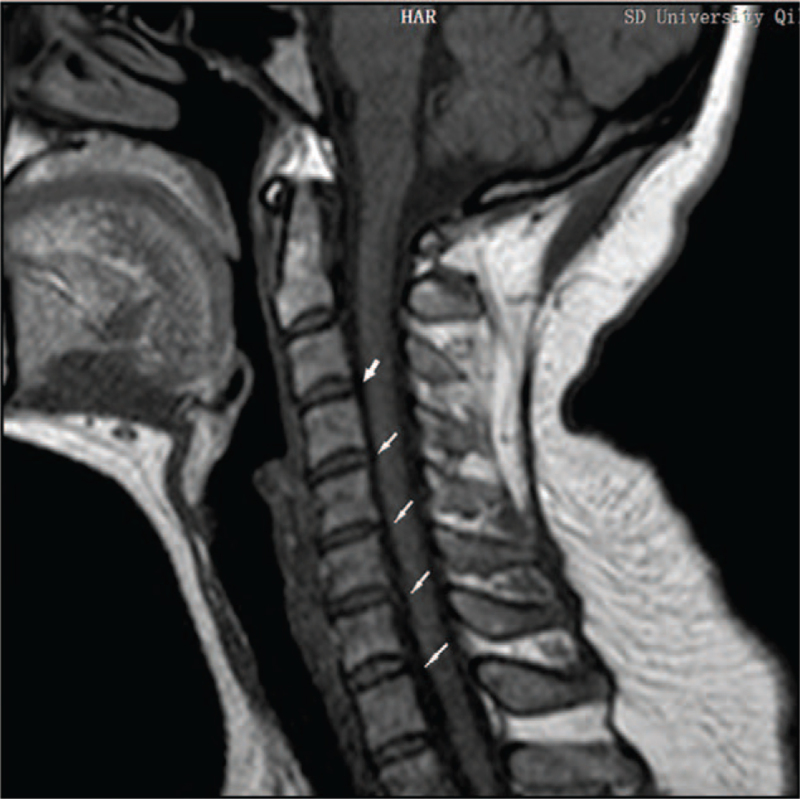
T1 weighted sagittal image of cervical spine after treatment. Note: White arrows indicate a reduction in cervical disc herniation and a significant reduction in spinal cord and dural compression.

**Figure 4:**
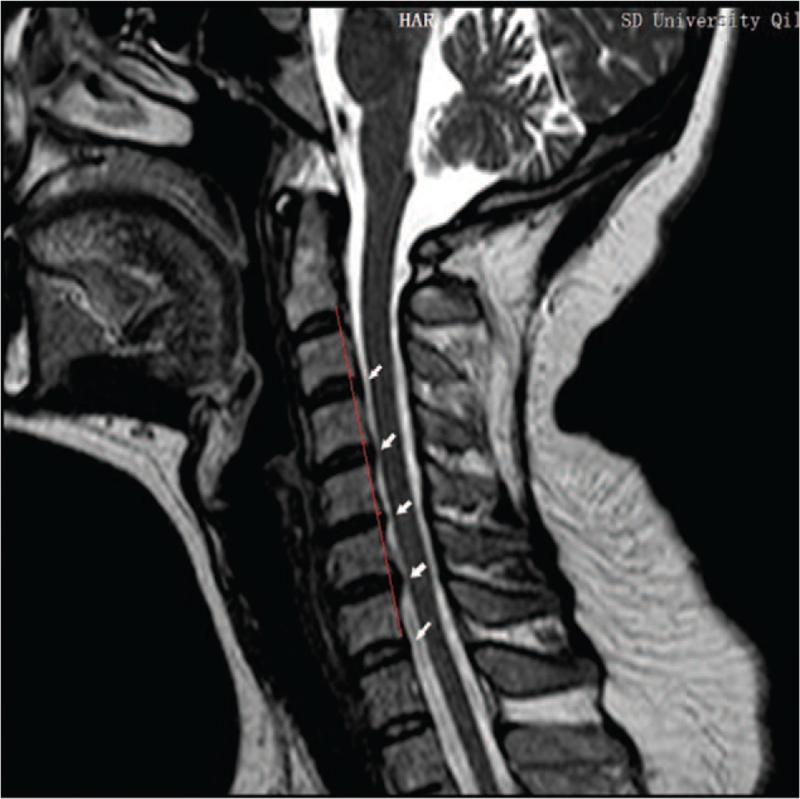
T2 weighted sagittal image of cervical spine after treatment. Note: The white arrows indicate a reduction in cervical disc herniation and a significant reduction in spinal cord and dural compression; the red line indicates the flexion angle of the cervical spine.

Physical examination (PE) and auxiliary inspection: Tingling of the left upper extremity, electrical symptoms, and radiation to the 4th and 5th fingers of the left hand. The physiological curvature of the cervical vertebra became straight and the degree of activity decreased (Table 1). Neck muscle edema and hypertrophy, high neck and shoulder muscle tension, C2–C7 acantha and paraspinal muscle tenderness (+), neck rotation test (±), percussion test (±); other tests were negative. MRI showed a C3–C7 disc herniation with C5–C7 spinal canal stenosis and cervical degeneration. The range of motion of the cervical spine in the sitting position (Table 1), and the grip strength of both hands was measured (Table 1). Milestones related to diagnoses and interventions Table 1 and Figures 1–4.

**Table 1:** Cervical active ROM evaluation, VAS score of neck pain, CCI and Grip strength evaluation before treatment and after treatment.

			Right		Left	
	Initial	After treatment	Initial	After treatment	Initial	After treatment
Rotation ROM			20°	45°	20°	45°
Lateral Flexion ROM			15°	45°	15°	45°
Flexion ROM	10°	42°				
Extension ROM	30°	40°				
VAS pain score	8	0				
CCI	−16.04%	−3.50%				
Grip strength			20.1 kg	22.6 kg	7.5 kg	19.2 kg
3 trials			19.6 kg	22.6 kg	6.8 kg	18.9 kg
			19.7 kg	21.2 kg	6.8 kg	18.9 kg

Diagnostic Assessment: The diagnosis was supported by clinical symptoms, PE, laboratory testing (measurement of cervical flexion muscle strength), and imaging. We can eliminate brain and cerebrovascular diseases. The patient had no financial, language, or cultural difficulties in diagnosis. The other diagnoses included multilevel cervical disc herniation. The patients were evaluated by the rehabilitation group; they could be treated with SDS combined with EA, and the prognosis was good.

Intervention methods: Physiotherapy did not require ethical approval. SDS (SpineMED US Patent# 7201729) was used for mechanical traction of the cervical spine under computer control. In the supine position, the patient lay flat on the SDS treatment bed, and the head and neck were fixed on the cervical vertebral retractor at the head of the SDS treatment bed. The parameters were set to cervical disc herniation segments C3-4, C4-5, C5-6, and C6-7. Cervical flexion 25°, continuous traction for 60 seconds, 17 pounds, released to 10 pounds, relax for 30 second, for a total of 20 cycles. Each treatment lasted for 30 minutes, with a frequency of 5 times per week. The patient also received EA (Yingdi KWD-8081 pulse acupuncture treatment instrument, Changzhou, Jiangsu, China). The basic acupoints are Fengchi (GB20, bilateral), Jianjin (GB21, bilateral), Jiaji (EXB2, bilateral), and Dazhui (GV14, 1point)^[2].^ Methods: EA was administered once a day. In this case, the intervention did not change.

Outcomes and follow-up: The patient's symptoms improved markedly after 4 weeks of outpatient rehabilitation. The patient's VAS score for neck pain decreased from 8 points before treatment to zero points. Soreness and tingling of the left upper limb and hand also disappeared. Her neck activity returned to normal, and her left-hand grip strength increased (Table 1). Re-examination of cervical MRI showed that the physiological curvature of the cervical spine was significantly improved, and the herniated disc was significantly improved; cervical curvature index changed from −16.04% (before treatment) to −3.50% (after treatment) (Figs. 1–4). The patient's symptoms disappeared, and her normal life function was restored. The patient was followed up in the sixth months and 1 year after treatment, and the patient's condition was stable without aggravation. Compliance and tolerance of the intervention were evaluated based on the patients’ pain levels.

Adverse and unanticipated events: During the first traction of the patient with SDS treatment for 15 minutes, there was an uncomfortable reaction to cervical pain and head burning. The traction treatment was terminated, and body temperature was measured. There was no significant change in body temperature within 15 minutes. The discomfort in the head and neck disappeared after 10 minutes of rest. No adverse reactions were noted. No adverse reactions were observed during the treatment of cervical spondylopathy by EA.

## 3 Discussion

This was a typical case of mixed cervical spondylosis caused by a herniated cervical disc. Cervical spine MRI showed that the physiological curvature of the cervical spine was straightened or even counterattacked. Multisegment intervertebral discs protruded and compressed the dural sac. At the same time, there were clinical symptoms, such as nerve root compression. The mechanism of SDS in the treatment: SDS traction can be directly (by reducing the volume of the hernia, creating negative pressure in the intervertebral disc, opening the intervertebral foramen space),^[3]^ indirectly (by relaxing paraspinal muscles,^[4]^ or by stretching the joint capsule or other supporting structures^[5]^ to relieve pain), to reduce the mechanical compression caused by intervertebral disc lesions. SDS traction is associated with pain relief and increased intervertebral disc height, which may be mediated, at least in part, by the recovery of intervertebral disc height.^[5]^ Owing to multilevel cervical disc protrusion, traction treatment is difficult. During traction, a series of pathophysiological reactions may occur due to mechanical friction and compression of the cervical nerve root. Thus, abnormal sensations of neck pain and head burning occurred, but the temperature test results were normal. Subsequently, the treatment should be stopped.

Studies have shown that EA is more effective than acupuncture alone in the treatment of neck pain caused by cervical spondylosis.^[2,6]^ EA-simulated exercise promotes skeletal muscle gene expression and regeneration.^[7]^ In EA therapy, in addition to the initial manual stimulation to find the correct needle position,^[8]^ electrical stimulation is usually applied to 2 adjacent needles for 15 to 20 minutes. These 2 techniques activate afferent nerve fibers, but the obvious difference lies in the increase in sympathetic nerve activity and repeated prolonged muscle contraction caused by EA, which is similar to the effect of physical exercise.^[9]^ Recent research has shown that EA can improve the biomechanical load of the cervical intervertebral disc joint cartilage^[10]^ and relieve pain symptoms.

The combination of SDS traction and EA not only corrects the biomechanics of cervical vertebrae but also strengthens the stability of the cervical vertebrae, and the symptoms of cervical spondylosis disappear. In summary, SDS combined with EA has a significant effect on patients with multisegment cervical disc herniation. This case report helps us choose appropriate physical therapy. The proposed method is simple, practical, and effective.

Limitations: This is a successful case of mixed cervical spondylosis caused by physiotherapy. Whether the intervention method is effective in this kind of crowd (cervical spondylosis caused by multi-level cervical disc herniation) may be used in clinical trial research.

## 4 Conclusion

SDS traction combined with EA can effectively improve the symptoms of cervical disc herniation, promote neuromuscular tissue edema and inflammatory absorption, improve and restore the physiological curvature of the cervical vertebrae, and reduce intervertebral disc pressure. It helps restore and rebuild the biomechanical balance of the cervical vertebrae without causing side effects. The prognosis for this disease is good.

## Author contributions

**Conceptualization:** Qing Xu.

**Data curation:** Qing Xu.

**Formal analysis:** Qing Xu.

**Methodology:** Qing Xu, Xintong Bao, Dongren Liu.

**Project administration:** Qing Xu.

**Visualization:** Xuewen Tian.

**Writing – original draft:** Qing Xu.

**Writing – review & editing:** Xuewen Tian, Qiangsan Sun.

## References

[1] Palejwala SK Rughani AI Dumont TM. Increased utilization of cervical disk arthroplasty in university hospitals with regional variation and socioeconomic discrepancies. World Neurosurg 2017;99:433–8. 27993738 10.1016/j.wneu.2016.12.016

[2] Wan BJ Huang W Zhang YX Zhang HS. Influence of electroacupuncture with penetration needling method on comprehensive pain score in pati ents with cervical spondylotic radiculopathy. Zhongguo Zhen Jiu 2013;33:407–10. 23885612

[3] Gay RE Ilharreborde B Zhao KD Berglund LJ Bronfort G An KN. Stress in lumbar intervertebral discs during distraction: a cadaveric study. Spine J 2008;8:982–90. 17981092 10.1016/j.spinee.2007.07.398 PMC2613278

[4] Letchuman R Deusinger RH. Comparison of sacrospinalis myoelectric activity and pain levels in patients undergoing static and in termittent lumbar traction. Spine (Phila Pa 1976) 1993;18:1361–5. 8211369 10.1097/00007632-199308000-00017

[5] Apfel CC Cakmakkaya OS Martin W. Restoration of disk height through non-surgical spinal decompression is associated with decreased discogenic low back pain: a retrospective cohort study. BMC Musculoskelet Disord 2010;11:155. 20615252 10.1186/1471-2474-11-155 PMC2912793

[6] Chen ZH Liang FR Yang MX Li DH Zhang Y Ren YL. Effect and safety of CX-DZ-II intelligent electroacupuncture therapeutic instrument for neck pain caused by cervical spondylos: study protocol for a randomized controlled trial. Chin J Integr Med 2020;26:375–81. 31372917 10.1007/s11655-019-3038-2

[7] Yan M Wang R Liu S. The mechanism of electroacupuncture at zusanli promotes macrophage polarization during the fibrotic process in contused skeletal muscle. Eur Surg Res 2019;60:196–207. 31694021 10.1159/000503130

[8] Langevin HM Schnyer R MacPherson H. Manual and electrical needle stimulation in acupuncture research: pitfalls and challenges of heteroge neity. J Altern Complement Med 2015;21:113–28. 25710206 10.1089/acm.2014.0186 PMC4855731

[9] Stener-Victorin E Jedel E Janson PO Sverrisdottir YB. Low-frequency electroacupuncture and physical exercise decrease high muscle sympathetic nerve activit y in polycystic ovary syndrome. Am J Physiol Regul Integr Comp Physiol 2009;297:R387–95. 19494176 10.1152/ajpregu.00197.2009

[10] Shi X Yu W Wang T. Electroacupuncture alleviates cartilage degradation: improvement in cartilage biomechanics via pain r elief and potentiation of muscle function in a rabbit model of knee osteoarthritis. Biomed Pharmacother 2020;123:109724. 31918209 10.1016/j.biopha.2019.109724

